# Short-term omalizumab as a potential rescue therapy for alopecia areata flares during Janus kinase inhibitor maintenance

**DOI:** 10.1016/j.jdin.2026.03.006

**Published:** 2026-03-23

**Authors:** Qin Wang, Ke Cheng, Jui-Ming Lin, Ke Tao, Minghuan Xu, Xinyi Zhang, Sichong Tang, Chunya Ni, Wenyu Wu, Jinran Lin

**Affiliations:** a Department of Dermatology, Huashan Hospital, Shanghai Institute of Dermatology, State Key Laboratory of Molecular Engineering of Polymers, Fudan University, Shanghai, China; b Department of Dermatology, Jing'an District Central Hospital, Shanghai, China; c Academy for Engineering and Technology, Fudan University, Shanghai, China

**Keywords:** allergy, alopecia areata, Janus kinase inhibitors, omalizumab, treatment flare

*To the Editor:* Alopecia areata (AA) is a chronic autoimmune disease for which oral Janus kinase (JAK) inhibitors have demonstrated robust and generally well-tolerated efficacy. However, long-term control is not fully durable, and flares can occur even in initial responders who remain on therapy.^1^^,^^2^ Despite this, the clinical profile of patients prone to such flares and effective management strategies remain unclear. In patients with an allergic background, therapies targeting Th2/allergic pathways have been associated with reduced relapse.^3^ We therefore characterized AA patients who relapsed despite continued JAK inhibitor therapy and evaluated short-term omalizumab as a rescue strategy during maintenance JAK inhibitor therapy.

We retrospectively analyzed 9 AA patients who developed flares during ongoing JAK inhibitor therapy. Demographic and clinical characteristics are summarized in Table I. All patients had an atopic background, and 7 of 9 (77.8%) showed elevated serum immunoglobulin E (IgE) levels (upper limit of normal: 60.0 KUA/L), with a mean IgE level of 158.1 (SD, 121.3) KUA/L. Clinical subtypes included patchy AA (*n* = 2, 22.2%), acute diffuse AA (*n* = 2, 22.2%), alopecia universalis/totalis (*n* = 4, 44.4%), and ophiasis (*n* = 1, 11.1%).

**Table I:** Demographic and clinical characteristics of patients

Characteristic	(*N* = 9)
Age, years, mean (SD)	23 (10.2)
Female, *n* (%)	4 (44.4%)
Weight, kg, mean (SD)	65.6 (12.9)
Time since onset of AA, mo, median (IQR)	36 (16-54)
Duration of current AA episode, mo, mean (SD)	18.8 (22.3)
Alopecia subtypes, *n* (%)	
Alopecia areata multiplex	2 (22.2%)
Diffuse alopecia areata	2 (22.2%)
Alopecia totalis or alopecia universalis	4 (44.4%)
Ophiasis alopecia	1 (11.1%)
Baseline severity of alopecia areata, *n* (%)	
Severe (SALT score 50-94)	5 (55.5%)
Very severe (SALT score 95-100)	4 (44.4%)
Eyebrow involvement, *n* (%)	2 (22.2%)
Eyelash involvement, *n* (%)	2 (22.2%)
Nail involvement, *n* (%)	1 (11.1%)
Comorbidity, *n* (%)	
Atopic dermatitis	5 (55.5%)
Chronic urticaria	5 (55.5%)
Cheilitis	1 (11.1%)
IgE level, KUA/L, mean (SD)	158.1 (121.3)
Co-administered JAK inhibitors, *n* (%)	
Baricitinib 4 mg once daily	2 (22.2%)
Upadacitinib 15 mg once daily	7 (77.8%)
Dose of omalizumab	300 mg at W0 and W4

*AA*, Alopecia areata; *IgE*, serum immunoglobulin E; *n*, number; *SALT*, Severity of Alopecia Tool; *SD*, standard deviation.

At baseline, the median SALT score was 90 (IQR, 80-95). All patients had previously achieved marked improvement with baricitinib 4 mg or upadacitinib 15 mg, reaching a median SALT score of 3 (IQR, 2-5) prior to relapse. All subsequently developed acute flares, defined as an increase in SALT score ≥10, without changes in JAK inhibitor dosage. Each patient received 2 doses of omalizumab 300 mg, administered at the time of flare (week 0, W0) and again at week 4 (W4) (Fig 1). The median SALT score at W0 was 20 (IQR, 15-35) and showed no statistically significant change at W4 (median, 15; IQR, 10-20). By week 12 (W12), the median SALT score had decreased to 9 (IQR, 5-15; Wilcoxon signed-rank test, *P* < .05 vs W0). SALT scores remained stable at weeks 16 and 24, with no evidence of rebound (Supplementary Fig 1, available via Mendeley at https://data.mendeley.com/datasets/t5yjfn8zv6/2). Representative clinical photographs are shown in Supplementary Fig 2, available via Mendeley at https://data.mendeley.com/datasets/t5yjfn8zv6/2. No new adverse events were observed during omalizumab treatment.

**Fig 1:**
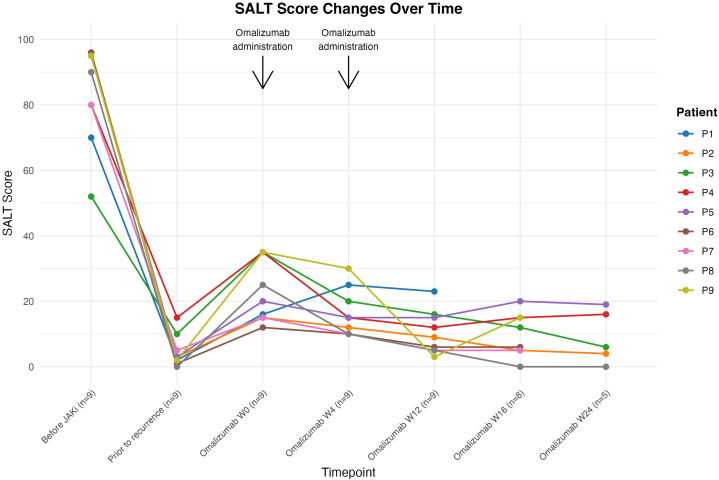
Changes in SALT scores in alopecia areata patients with acute flares during ongoing JAK inhibitor therapy following omalizumab administration. *JAK*, Janus kinase; *SALT*, Severity of Alopecia Tool.

Although AA has traditionally been regarded as a Th1-dominant disorder, accumulating evidence supports a clinically relevant Th2 component. Allergic inflammation has been implicated in the onset and relapse of AA, and elevated IgE levels have been associated with increased disease severity and poorer treatment responsiveness.^4^ Within this immunological context, we observed that disease flares during ongoing JAK inhibitor therapy in our cohort occurred predominantly in patients with atopy and elevated IgE. Omalizumab, by neutralizing IgE and suppressing downstream mast-cell and eosinophil activation, may attenuate this Th2-driven pathway.^5^ The delayed but sustained improvement observed from week 12 onward suggests a gradual immunomodulatory effect rather than rapid suppression. Clinically, these findings suggest that short-term omalizumab may be a rescue option for AA flares during JAK inhibitor therapy, especially in patients with atopic predisposition and elevated IgE. Key limitations of this study include its retrospective design and small sample size.

### Declaration of generative AI and AI-assisted technologies in the writing process

AI was used solely for language polishing and grammar checking. No content generation, data analysis, or scientific interpretation was performed.

## Conflicts of interest

None disclosed.
